# A Co-Occurrence of Serologically Proven Myasthenia Gravis and Pharyngeal-Cervical-Brachial Variant of Guillain-Barré Syndrome

**DOI:** 10.1155/2019/4695010

**Published:** 2019-04-08

**Authors:** Stacey Ho, Antonio Liu

**Affiliations:** Ross University School of Medicine, Department of Neurology, California Hospital Medical Center, Los Angeles, California, USA

## Abstract

We report on a co-occurrence case of ocular myasthenia gravis with exacerbation leading to myasthenic crisis in addition to pharyngeal-cervical-brachial variant of Guillain–Barré syndrome in a patient with severe oropharyngeal dysphagia and acute respiratory failure.

## 1. Introduction

Myasthenia gravis (MG) is an autoimmune disease of the neuromuscular junction with a prevalence of approximately 20 per 100,000 in the US and between 15 and 179 per million worldwide [1, 2]. In patients less than 40 years of age, female to male ratio is 3:1. Patients between 40 and 50 years show an equal ratio between female and male. However, males over the age of 50 are more likely to have MG than their female counterpart [2]. MG causes fluctuating weakness that worsens with exertion and progresses throughout the day [1]. Approximately two-thirds of the patients with MG initially have extrinsic ocular muscles (EOMs) involvement that progresses to involve other bulbar muscles and limb musculature with the eventual presentation of generalized myasthenia gravis (gMG) [2]. In approximately 10% of MG patients, symptoms are limited to only EOMs causing the condition to be known as ocular MG (oMG) [2].

Guillain-Barré syndrome (GBS) is a fulminant polyradiculoneuropathy that is characterized by acute ascending weakness that is usually severe, and autoimmune-related leading to generalized paralysis [3]. The worldwide incidence of GBS is approximately 0.6–2.4 cases per 100,000 annually, with increased incidence with age and slightly more predominance in males [3, 4]. The etiology of GBS is believed to be autoimmune, in which the majority of cases (70%) showed upper respiratory infections that led to the stimulation of antiganglioside antibodies production [3]. Patients usually show signs and symptoms 1–3 weeks after acute infectious process, usually respiratory or gastrointestinal, that include the follow organisms:* Campylobacter jejuni*,* Mycoplasma pneumonia*,* Haemophilus influenzae*, cytomegalovirus, Epstein-Barr virus, and influenza [3, 4].

Although both MG and GBS present with weaknesses, the concurrent development of MG and GBS is rare [5]. A recent study found 13 cases of concurrent MG and GBS between 1982 and 2016 [6]. It has been suggested that concurrent development is due to molecular mimicry between infectious agents and self-antigens [5]. These antibodies may have shown cross-reactions against both myelin proteins of peripheral nerves and acetylcholine receptors of neuromuscular junctions; and thymoma-associated multiorgan autoimmunity may also play a factor in initiating autoimmune process [5].

## 2. Objective

We report on a co-occurrence case of ocular myasthenia gravis with exacerbation leading to myasthenic crisis in addition to pharyngeal-cervical-brachial variant of Guillain–Barré syndrome in a patient with severe oropharyngeal dysphagia and acute respiratory failure.

## 3. Case Report

An 83-year-old Hispanic male with an extensive past medical history including chronic hepatitis C, coccidioidomycosis, ocular myasthenia gravis, and interstitial lung disease presented to the Emergency Department (ED) with a complaint of chronic cough, shortness of breath, upper extremities weakness with diplopia, and dysphagia. The patient reported a daily cough with sputum production since Thanksgiving 2017 and became more severe in the past 3 days. He denied any weakness in his lower extremities and reported that he was able to garden up until 3 months ago when he started having upper extremities weakness. In the ED, the patient's SPO2 was 94%, initial chest X-ray showed no acute pathology, and CT of the chest showed no signs of thymoma or any other lung pathologies. He was admitted to telemetry with a BiPAP for further evaluation. The patient's Video Swallow Study showed severe oropharyngeal dysphagia. We were consulted as the patient had severe dysphagia and a history of ocular myasthenia gravis. However, the patient developed acute respiratory failure and was intubated before an assessment and evaluation can be performed.

Upon review of the patient's medical record, it showed that he was diagnosed with myasthenia gravis in 2017 when he was found to have AChR binding antibodies of 40.70 nmol/L, AChR blocking antibodies of 56%, and AChR modulating antibodies of 54%. He was prescribed pyridostigmine 60mg 1 tablet three times daily as treatment; however, his adherent to treatment regime was questionable; and he has had recurring follow-ups with his primary care physician for ocular myasthenia gravis. The patient's lab result in the ED showed AChR binding antibodies of 276 nmol/L, AChR blocking antibodies of 75%, and AChR modulating antibodies of 91%, all of which displayed a major increase in antibodies compared to his results at time of diagnosis. Due to the patient's history of ocular myasthenia gravis, his current state of severe dysphagia, and acute respiratory failure, we ordered a serum anti-GBS antibody work-up for our patient when he showed signs of improvement from diplopia but his respiratory status remained the same. The results revealed that the patient had GM1 antibodies of 81 units and GD1b antibodies of 52 units, which are serology markers seen in GBS. The patient was started on IVIG and methylprednisolone while continuing with pyridostigmine. Several days later, the patient exhibited significant improvement in respiratory status and clinical signs of improved swallow function, and his diplopia has been resolved.

## 4. Discussion

Myasthenia gravis is an autoimmune disease of the neuromuscular junction that presents with fluctuating pronounced exertional voluntary muscle weakness [7]. While Guillain-Barré syndrome and its variants are a group of autoimmune disease of the proximal peripheral nerves and nerve roots mediated by lymphocytic mononuclear cell infiltration and macrophage-associated segmental demyelination that is associated with limb weakness and areflexia [8].

Diagnosing MG is based on clinical history and neurologic examination and is confirmed by electrodiagnostic testing and the presence of serum autoantibodies directed against proteins—acetylcholine receptor (AChR) and muscle-specific tyrosine kinase (MuSK)—at the neuromuscular junction [1]. MG can be further divided into subtypes that includes (1) early-onset MG with age of onset <50 years, usually females, and has thymic hyperplasia, (2) late-onset MG with age of onset >50 years, usually males, and has thymic atrophy, (3) thymoma-associated MG, (4) MG with the presence of anti-MuSK antibodies, (5) oMG, and (6) MG with no detectable AChR and MuSK antibodies [2]. However, patients may present with overlapping of subtypes. The majority of patients with gMG (85%) and oMG (50%) will have anti-AChR antibodies, with an additional 8%-10% of those with gMG having anti-MuSK antibodies [1]. MG with evidence of thymoma is almost always detected to have anti-AChR antibodies; and it may also have additional paraneoplasia-associated antibodies, such as antivoltage-gated K^+^ and Ca^++^ channels, anti-Hu, antidihydropyrimidinase-related protein 5, and antiglutamic acid decarboxylase antibodies [2]. In 40% of patients with no detectable anti-AChR antibodies, anti-MuSK and another postsynaptic neuromuscular junction (NMJ) protein are detected [2]. These patients have atypical clinical presentations, such as selective facial, bulbar, neck, or respiratory muscle weakness with occasional marked muscle atrophy and relative sparing of the ocular muscles [2]. Other MG patients can present with seronegative MG that lacks both anti-AChR and anti-MuSK antibodies.

Although our patient presented with an increase in AChR titers during myasthenic crisis, studies have shown that the level of AChR antibodies does not relate with the clinical severity of MG. A study done by Aurangzeb et al. showed that out of the 71 seropositive MG patients in their study, 57.7% of the patients had low titers and 42.2% had high titers [9]. And while the majority of those patients exhibited Osserman's stage III (acute severe generalized disease with respiratory failure), there was no correlation between AChR antibodies titers and Osserman's classification [9].

Treatment for MG is immunosuppressive therapies, such as prednisone, azathioprine, cyclosporine, and mycophenolate mofetil [1]. Cholinesterase inhibiting agents typically only provide temporary relief from symptoms without altering the course of MG [1]. Thymectomy is usually beneficial in those with evidence of thymoma on chest CT [1]. Plasmapheresis and IV immunoglobulin (IVIG) are usually reserved for patients who encounter a myasthenic crisis [1].

The presentation of GBS include areflexia and limb weakness and rarely sensory loss proceeding to neuromuscular paralysis involving bulbar, facial, and respiratory function with maximum severity of symptoms in 2–4 weeks [3]. Patients typically presents initially with lower and upper extremities weakness (32%) or selective proximal and distal lower extremities weakness (56%) that often spreads to the arm while some have onset of weakness in the upper extremities (12%) [8]. Lower extremities are usually affected more often than upper extremities, and facial diparesis occurs in 50% of patients [3]. The weakness exhibited in GBS is typically seen in a pyramidal distribution with ankle dorsiflexion and knee and hip flexion often severely affected, while weakness in the arms is usually more severe in shoulder abduction and elbow extension [4]. Lumbar pain is also common and may represent inflammation in the nerve roots and breakdown in the nerve CSF barrier that leads to protein leaking into the CSF [4]. Respiratory involvement may be acute with vital capacity falling steadily, and intubation and ventilation are required at level of approximately 1 liter [4]. Studies have shown that mortality in GBS is between 5% and 10%, and 60% of patients who recover are able to walk unaided by 12 months [4].

Patients with GBS may present with clinical variants based on the types of nerve fibers involved (motor, sensory, and cranial or autonomic), predominant mode of fiber injury (demyelinating versus axonal), and the presence of alteration of consciousness [8]. In a study done by Koga et al., IgG anti-GT1a and anti-GM1b were particularly valuable in diagnosing GBS and its variants in which early appearance of bulbar involvement was observed [10]. The major gangliosides GM1 and GD1a and the minor ones GM1b, GalNAc-GD1a, and GT1a are target molecules for autoantibodies found in GBS and its variants [10].

Pharyngeal-cervical-brachial (PCB) variant of Guillain-Barré syndrome presents in 3% of cases and causes rapidly progressive oropharyngeal and cervicobrachial weakness associated with areflexia in the upper extremities without ophthalmoplegia or lower extremities weakness [11]. Motor and strength of lower extremities are typically preserved, thus making PCB a localized subtype of GBS that is a form of acute motor axonal neuropathy (AMAN) [11]. Autoantibodies that are associated with PCB include IgG anti-GM1, anti-GD1a, anti-GD1b, and anti-GT1a [11]. Our patient had serum antibodies showing GM1 antibodies of 81 units and GD1b antibodies of 52 units and presented with clinical symptoms of a pure motor bulbar palsy typically seen in PCB. Although anti-GD1b is commonly seen in cases of GBS with ataxia and is usually associated with sensory neuropathy [12], both ataxia and sensory neuropathy presentation were not observed in our patient.

Treatment of GBS includes admission to intensive care with respiratory support when required and early recognition of respiratory failure [13]. Patients with severe dysphagia may require nasogastric and feeding tubes, while intubation should be considered for patients who cannot tolerate their secretions or who have an ineffective cough [8]. Treatment with pain modulating drugs such as antidepressants, gabapentin, pregabalin, carbamazepine, tramadol, and mexiletine in patient with marked radicular back pain or neuropathic pain refractory to acetaminophen or NSAIDs [13]. Patients with weakness impairing function or any respiratory involvement should be treated with plasmapheresis or IVIG [13].

In summary, the diagnosis of PCB can be challenging due to its overlapping features with other variants of GBS. A history of prior respiratory tract infection (as seen in our patient) or diarrheal illness is common in GBS and can be often overlooked. The rate of disease progression can also provide clues and is typically acute with steady progression [11]. Particularly, PCB should be differentiated from MG patients who display early or prominent ocular-bulbar weakness, which may be associated with anti-MuSK in the absence of anti-AChR antibodies. In our case, the patient was already diagnosed with MG and presented with myasthenic crisis. Furthermore, our patient showed signs of severe bulbar palsy that required further investigation. Thus, a good history and neurological findings can help differentiate PCB from other causes of orpopharyngeal and cervicobrachial weakness [11].
